# The expression of pregnancy-associated plasma protein-A (PAPP-A) in human blastocoel fluid–conditioned media: a proof of concept study

**DOI:** 10.1007/s10815-022-02393-4

**Published:** 2022-01-11

**Authors:** Shahryar K. Kavoussi, Shu-Hung Chen, John David Wininger, Arnav Lal, William E. Roudebush, Hayes C. Lanford, Amy S. Esqueda, Maya Barsky, Dan I. Lebovic, Parviz K. Kavoussi, Melissa S. Gilkey, Justin Chen, Graham L. Machen, Renee J. Chosed

**Affiliations:** 1 Austin Fertility & Reproductive Medicine/Westlake IVF, 300 Beardsley Lane, Bldg B, Suite 200, Austin, TX 78746 USA; 2 Department of Biomedical Sciences, University of South Carolina School of Medicine Greenville, Greenville, SC 29605 USA

**Keywords:** Blastocoel fluid, Blastocyst, PAPP-A, Pregnancy-associated plasma protein-A, IVF

## Abstract

**Purpose:**

The aim of this study was to determine if pregnancy-associated plasma protein-A (PAPP-A), typically measured in maternal serum and a potential predictor of adverse maternal and fetal outcomes such as spontaneous miscarriage, pre-eclampsia, and stillbirth, is expressed in blastocoel fluid–conditioned media (BFCM) at the embryonic blastocyst stage.

**Design:**

This is an in vitro study.

**Methods:**

BFCM samples from trophectoderm-tested euploid blastocysts (*n* = 80) from in vitro fertilization/intracytoplasmic sperm injection (IVF/ICSI) patients were analyzed for PAPP-A mRNA. BFCM was obtained from blastocyst stage embryos in 20 uL drops. Blastocysts underwent trophectoderm biopsy for preimplantation genetic testing for aneuploidy prior to blastocyst vitrification and BFCM collection for snap freezing. cfDNA was synthesized using BFCM collected from 80 individual euploid blastocysts. Next, real-time qPCR was performed to detect expression of PAPP-A with GAPDH for normalization of expression in each sample.

**Results:**

PAPP-A mRNA was detected in 45 of 80 BFCM samples (56.3%), with varying levels of expression across samples.

**Conclusion:**

Our study demonstrates the expression of PAPP-A in BFCM. To our knowledge, this is the first study to report detection of PAPP-A mRNA in BFCM. Further studies are required and underway to investigate a greater number of BFCM samples as well as the possible correlation of PAPP-A expression with pregnancy outcomes of transferred euploid blastocysts. If found to predict IVF and obstetric outcomes, PAPP-A may provide additional information along with embryonic euploidy for the selection of the optimal blastocyst for embryo transfer.

## Introduction

There is a great need for early detection and prevention of adverse maternal and fetal prenatal outcomes. If a reliable marker can be used, as early as the embryonic stage for in vitro fertilization (IVF) cases, to predict the likelihood of such outcomes, then this process could be referred to as preimplantation prenatal screening (PPS). Pregnancy-associated plasma protein A (PAPP-A), predominantly produced by placental trophoblastic cells [1] and measured in maternal serum during the first trimester for clinical purposes, has been shown to be a potential predictor of adverse obstetric outcomes [2–6] such as spontaneous miscarriage, small for gestational age (SGA)/intrauterine growth restriction (IUGR), pre-eclampsia, and intrauterine fetal demise (IUFD) [1, 7]. Furthermore, it has been suggested that a first trimester screening result of PAPP-A less than 0.4 multiples of the median (MoM) in chromosomally and morphologically healthy fetuses may correlate with placental-mediated pregnancy complications [7–9].

PAPP-A is a 4 kilodalton dimeric metalloproteinase that regulates insulin-like growth factor (IGF)-I and IGF-II activity [10] and cleaves IGF-binding protein-4. Since the time that it was first detected in the serum plasma of pregnant women [11], maternal serum PAPP-A has been the subject of many publications, and investigators have studied the secretion of PAPP-A by trophoblastic cells as well [1, 12]. Recent data suggest that low levels of PAPP-A in maternal serum is associated with abnormal placentation which may explain potential downstream maternal and fetal complications [13]. A recent retrospective study reported the interesting finding of a significant, positive association between increased first trimester serum PAPP-A levels and placenta accreta [14]. Although existing data indicate that PAPP-A is expressed at the level of the human ovary [15], specifically in granulosa cells, theca cells [16, 17], follicular fluid [10], and in cumulus granulosa cell masses [18], as well as downstream in trophoblastic cells, there is lack of data regarding the potential expression of PAPP-A at the human embryonic level in vitro.

Blastocoel fluid (BF) has been found to be a potential source of noninvasive testing for genomic, transcriptomic, proteomic, and metabolomic markers at the embryonic blastocyst stage [19–23]. In the search for reliable methods to karyotype preimplantation embryos in a manner which is less invasive than trophectoderm biopsy of blastocyst stage embryos, previous literature has demonstrated the presence of cell-free DNA (cfDNA) in BF and correlation with embryonic morphology has been shown as well [24]. Furthermore, cell-free nucleic acid (cfNA) content in human BF–conditioned media (BFCM) may provide additional assessment of embryo implantation potential [25].

PAPP-A expressed in human villi tissues promotes the proliferation of trophoblast cells and cell adhesion in an autocrine manner in vitro [1, 26]. Furthermore, in vivo, PAPP-A blockade via PAPP-A antibody injection into the uterine cavity suppressed the embryo implantation rate compared with an injection control group in a pregnant murine model [1]. The question has arisen as to whether PAPP-A is expressed at the blastocyst stage of embryonic development. If a marker such as PAPP-A were to be expressed in BFCM at the blastocyst stage of embryonic development and if further study were to show that PAPP-A predicts pregnancy results of euploid embryo transfer as well as potential downstream maternal and fetal obstetric outcomes, PAPP-A may serve as an adjunct to the selection of the optimal embryo for transfer. The aim of this study was to determine if PAPP-A is expressed in BFCM in vitro.

## Materials and methods

BFCM was obtained from blastocyst stage embryos following standard, routine-controlled ovarian stimulation and subsequent IVF processes. In brief, female patients had undergone their planned routine IVF cases, which consisted of controlled ovarian stimulation with exogenous gonadotropins, the use of gonadotropin hormone antagonist for suppression of luteinizing hormone prior to trigger with leuprolide acetate, and/or recombinant human chorionic gonadotropin (hCG) for final oocyte maturation prior to transvaginal ultrasound-guided oocyte retrieval 35 h later. Oocytes were isolated, and intracytoplasmic sperm injection (ICSI) was performed to achieve in vitro fertilization, with culture of embryos to the blastocyst stage of embryonic development by day 5 and day 6 of embryo culture. Good quality blastocysts were considered to be those with a grade of 2BB or higher, in accordance with Gardner and Schoolcraft’s grading system for blastocysts [27]. All 80 of the blastocysts in this study had undergone trophectoderm biopsy for preimplantation genetic testing for aneuploidy (PGT-A) prior to blastocyst vitrification and BFCM collection.

Per routine embryology laboratory protocol, each blastocyst was placed in a 20 μL medium drop under oil, laser pulse was used to open the cellular junctions between trophectoderm cells, and trophectoderm biopsy was performed so that the biopsied cells could be sent to a reference genetics laboratory for PGT-A via next-generation sequencing. As the blastocyst collapsed, blastocoel fluid was extruded into the drop of medium and the blastocyst was removed from the medium drop for subsequent blastocyst vitrification. The medium drop containing BF was collected and mixed via pipetting. Each BFCM sample was subsequently stored at − 20 °C for further analysis.

Real-time quantitative polymerase chain reaction (RT-qPCR) was performed for PAPP-A. Prior to RT-qPCR, RNA content in BFCM from individual euploid preimplantation embryos was assessed using a RNA 6000 Pico Kit (Agilent) with an Agilent 2100 Bioanalyzer to determine if RNA was present in the BFCM samples. One microliter of each BFCM sample was diluted tenfold in nuclease-free water. The diluted samples were then assessed for the total RNA present using the RNA 6000 Pico Kit chip with the Bioanalyzer as per manufacturer’s instructions. Next, individual BFCM samples (undiluted) were treated with DNaseI (RNAse free, Thermo Fisher, USA) for 30 min at 37 °C followed by heat inactivation at 65 °C for 10 min. Samples were then subjected to cDNA synthesis (High-Capacity cDNA Reverse Transcription Kit, Applied Biosystems, USA) per manufacturer’s instructions. cDNA quantity was then determined with a High Sensitivity DNA Kit (Agilent) with an Agilent 2100 Bioanalyzer as per manufacturer’s instructions. Forty nanograms of cDNA template (for each BFCM sample) was then combined with 2X TaqMan Master Mix, 20X Gene Expression Assay (GAPDH and PAPP-A specific) and nuclease-free water per manufacturer’s instructions (Applied Biosystems). Duplicate reactions for each sample were run for each gene of interest using a 7500 Fast Real-Time PCR System (Applied Biosystems, USA) at 50 °C for 2 min, 95 °C for 20 s, followed by 40 cycles of 95 °C for 3 s and 60 °C for 30 s.

Negative ΔCt (calculated with PAPP-A C_t_ value normalized against GAPDH C_t_) led to determination of PAPP-A mRNA expression in each sample. This analysis made use of the threshold consolidation method since there was no true BFCM control 28 .

IRB exemption was obtained by St. David’s Institutional Review Board due to the de-identified nature of the data as well as the use of BFCM that routinely would be discarded at the time of collapsing blastocysts for vitrification. Statistical analyses of variables among subjects with PAPP-A mRNA in BFCM as compared with subjects without PAPP-A in BFCM were performed with *t*-tests. Statistical analyses of variables among blastocysts with PAPP-A in BFCM as compared with blastocysts without PAPP-A mRNA in BFCM were performed with chi-square tests, with *P*-value of < 0.05 for statistical significance.

## Results

A sample size of 80 trophectoderm-tested euploid day 5 (*n* = 70) and day 6 (*n* = 10) good quality blastocysts from 36 patients that underwent IVF/ICSI/PGT-A/freeze-all had BFCM analyzed for PAPP-A mRNA expression. Patient and embryonic characteristics are listed in Table 1. PAPP-A mRNA was detected in 45 of 80 BFCM samples (56.3%), with varying levels of expression. PAPP-A expression ranged from a minimum of onefold change ratio to a maximum of 250,326.9 fold change ratio, with a mean of 8,523.5 and a standard deviation of 36,694. Of note, the maximum value was an outlier which affected the mean and standard deviation values, as the second highest fold change ratio was 16,295.3. There were no differences in mean female age for the subjects with at least one BFCM sample with PAPP-A mRNA detected (*n* = 26) and those with no BFCM samples with detectable PAPP-A mRNA (*n* = 10) (36.1 years and 36.4 years, respectively; *P*-value = 0.39), mean AMH level (2.45 ng/mL and 3.45 ng/mL, respectively; *P*-value = 0.12), peak estradiol level (2209 pg/mL and 2310 pg/mL, respectively; *P*-value = 0.41), and total gonadotropin dose for COS (3614 IU and 3292 IU, respectively; *P*-value = 0.23) between groups as well. Of the 70 day 5 blastocysts in the study, 37 had PAPP-A mRNA detected in their BFCM samples, and of the 10 day 6 blastocysts in the study, 8 had PAPP-A mRNA detected in their BFCM samples (chi-square statistic with Yates correction = 1.63; *P*-value = 0.20). Of the 45 blastocysts that had PAPP-A mRNA detected in their BFCM samples, 36 blastocysts (80%) had trophectoderm grading of A and 9 blastocysts had trophectoderm grading of B; of the 35 blastocysts that had no detectable PAPP-A mRNA in their BFCM samples, 28 (80%) had trophectoderm grading of A and 7 blastocysts had trophectoderm grading of B (chi-square statistic with Yates correction = 0.08; *P*-value = 0.78). Of the 20 patients who had more than one trophectoderm-tested euploid blastocyst, 10 (50%) had a sibling blastocyst with PAPP-A expression.

**Table 1:** Characteristics and variables, including demographics and outcomes for study subjects. *t*-test performed for female age, AMH level, peak estradiol, and total gonadotropin dose (results are represented as means and standard deviations). Chi-square performed for number of day 5 blastocysts vs day 6 blastocysts as well as trophectoderm grade A vs trophectoderm grade B

	+ PAPP-A in BFCM	− PAPP-A in BFCM	*P* value
Number of subjects (*n* = 36)	**26 (72%)**	**10 (28%)**	
Mean female age in years	36.1 ± 3.1	36.4 ± 2.6	0.39
Mean AMH level (ng/mL)	2.45 ± 1.5	3.48 ± 3.7	0.12
Mean peak estradiol (pg/mL)	2209 ± 1197.1	2310 ± 1287.7	0.41
Mean total gonadotropin dose (IU)	3614 ± 866.3	3292 ± 1748.3	0.23
Number of blastocysts (*n* = 80)	**45 (56%)**	**35 (44%)**	
Number of day 5 blastocysts	37 (82%)	33 (94%)	0.20
Number of day 6 blastocysts	8 (18%)	2 (6%)	
Trophectoderm grade A	36 (80%)	28 (80%)	0.78
Trophectoderm grade B	9 (20%)	7 (20%)	

## Discussion

Although PAPP-A has previously been measured in vitro at the level of the human ovary, specifically in granulosa cells, theca cells [16, 17], follicular fluid [10, 15], and in cumulus granulosa cell masses [18], as well as downstream in trophoblastic cells, our study is the first study to report PAPP-A expression at the human blastocyst stage in vitro. For example, the presence of immunoreactive PAPP-A has been demonstrated in culture medium conditioned by human ovarian granulosa cells [29]. Furthermore, PAPP-A was found to be expressed in follicular fluid, with immunostaining having shown PAPP-A localized to the theca cell layer in small antral follicles of 4–6 mm diameter, with PAPP-A expression shifting inward to the granulosa cell layer as follicles mature in size and become pre-ovulatory [10]. In an investigation of the potential source of PAPP-A production in pregnancy, an in vitro study in 2003 reported the expression of PAPP-A mRNA in total placental extracts, and PAPP-A protein was detected in the cytoplasm of cytotrophoblast cells as well as syncytiotrophoblast cells, with greater expression with the latter cell type’s formation [12].

In the realm of IVF-conceived pregnancies, there are conflicting data regarding first trimester maternal serum PAPP-A levels as compared with pregnancies achieved without IVF. Some data suggest no difference in PAPP-A levels in pregnancies resulting from fresh embryo transfer (ET), FET, or spontaneous conception [30], and other studies have shown lower first trimester maternal serum PAPP-A levels in pregnancies conceived as a result of either fresh ET or FET as compared with spontaneous pregnancies [31–37], which may support the concept of suboptimal placentation leading to related pregnancy complications, particularly in cases of fresh embryo transfers. In a prospective observational study comparing levels of angiogenic markers and markers of early placentation in maternal serum among women who conceived via fresh versus frozen ET, women who achieved pregnancy via FET had higher serum levels of PAPP-A than those who achieved pregnancy via fresh ET, consistent with previous clinical data suggesting more favorable obstetric outcomes with FET than fresh ET [38]. Interestingly, one study showed no correlation between blastocyst morphology parameters and first trimester maternal serum PAPP-A levels in ongoing pregnancies, which were defined as pregnancies which progressed to at least beyond 13 weeks of gestation in that study [39]. There are data to suggest that peak E2 level on the day of trigger is not associated with low maternal serum PAPP-A levels in pregnancies that result from fresh ET [40] although another publication demonstrated lower maternal serum PAPP-A levels in IVF and ICSI pregnancies compared with non-IVF and ICSI pregnancies. Furthermore, in the latter study, a correlation was found between peak E2 level at triggering and low first trimester maternal serum PAPP-A in IVF pregnancies [41]. Placental volume of pregnancies achieved via FET has been shown to be greater than that of pregnancies resulting from fresh ET and spontaneous pregnancy, with a positive correlation having been observed between placental volume and first trimester maternal serum PAPP-A levels [42].

In the present study, the proof of concept that PAPP-A is expressed in BFCM of biopsied blastocysts was achieved. There are several possibilities as to why some BFCM samples had no detectable of PAPP-A mRNA, including no release of PAPP-A mRNA from the embryonic cells of some blastocysts or mRNA levels below the lower limit of detection. Limitations of our study include the small sample sizes of blastocysts and patients; however, data collection from larger sample sizes is underway in order to have a well-powered study to follow euploid FET and obstetric outcomes. Such studies are needed in order to address whether PAPP-A expression at the blastocyst stage impacts the efficiency of human embryonic implantation and downstream placental health, which in turn affects maternal and fetal outcomes.

## Conclusion

We have demonstrated the expression of PAPP-A in the BFCM of preimplantation blastocyst stage embryos in vitro. Larger prospective studies are necessary and underway to further assess whether PAPP-A expression at the level of human blastocyst stage embryo can predict maternal and fetal pregnancy outcomes of trophectoderm-tested euploid blastocyst transfer. If further study shows that PAPP-A in BFCM and/or biopsied embryonic cells predicts adverse obstetric outcomes, then this next level of embryo selection, in addition to euploid status, may optimize the potential for healthy live birth for IVF patients.
